# Respiratory Virus Infection and Risk of Invasive Meningococcal Disease in Central Ontario, Canada

**DOI:** 10.1371/journal.pone.0015493

**Published:** 2010-11-17

**Authors:** Ashleigh R. Tuite, Laura M. Kinlin, Stefan P. Kuster, Frances Jamieson, Jeffrey C. Kwong, Allison McGeer, David N. Fisman

**Affiliations:** 1 Dalla Lana School of Public Health, University of Toronto, Toronto, Canada; 2 Faculty of Medicine, Dalhousie University, Halifax, Canada; 3 Mount Sinai Hospital, Toronto, Canada; 4 Department of Health Policy, Management and Evaluation, University of Toronto, Toronto, Canada; 5 The Ontario Agency for Health Protection and Promotion, Toronto, Canada; 6 Department of Family and Community Medicine, University of Toronto, Toronto, Canada; 7 Institute for Clinical Evaluative Sciences, Toronto, Canada; The University of Hong Kong, Hong Kong

## Abstract

**Background:**

In temperate climates, invasive meningococcal disease (IMD) incidence tends to coincide with or closely follow peak incidence of influenza virus infection; at a seasonal level, increased influenza activity frequently correlates with increased seasonal risk of IMD.

**Methods:**

We evaluated 240 cases of IMD reported in central Ontario, Canada, from 2000 to 2006. Associations between environmental and virological (influenza A, influenza B and respiratory syncytial virus (RSV)) exposures and IMD incidence were evaluated using negative binomial regression models controlling for seasonal oscillation. Acute effects of weekly respiratory virus activity on IMD risk were evaluated using a matched-period case-crossover design with random directionality of control selection. Effects were estimated using conditional logistic regression.

**Results:**

Multivariable negative binomial regression identified elevated IMD risk with increasing influenza A activity (per 100 case increase, incidence rate ratio = 1.18, 95% confidence interval (CI): 1.06, 1.31). In case-crossover models, increasing weekly influenza A activity was associated with an acute increase in the risk of IMD (per 100 case increase, odds ratio (OR)  = 2.03, 95% CI: 1.28 to 3.23). Increasing weekly RSV activity was associated with increased risk of IMD after adjusting for RSV activity in the previous 3 weeks (per 100 case increase, OR = 4.31, 95% CI: 1.14, 16.32). No change in disease risk was seen with increasing influenza B activity.

**Conclusions:**

We have identified an acute effect of influenza A and RSV activity on IMD risk. If confirmed, these finding suggest that influenza vaccination may have the indirect benefit of reducing IMD risk.

## Introduction

*Neisseria meningitidis* is a leading cause of meningitis and bacteremia worldwide [1]. Invasive meningococcal disease (IMD) may be epidemic (e.g., in sub-Saharan Africa) or endemic (e.g., Canada and the United States). Infection occurs most commonly in infants and young children, with a second peak in incidence observed among adolescents and young adults [1], [2]. Incidence of IMD has declined in some regions recently [3], but the high case-fatality rates associated with infection [1] and the risk of transmission to contacts make IMD a disease of continuing public health importance.

Invasive meningococcal disease (IMD) displays marked seasonality; in temperate climates, IMD incidence tends to peak in late winter and early spring [1], [2]. As with other infectious diseases demonstrating seasonal periodicity, the mechanisms driving these fluctuations remain poorly understood. Environmental exposures, including relative humidity [4], [5], [6], [7], [8], [9] and ultraviolet radiation [9], may contribute to IMD seasonality, although these effects appear to be regionally specific in direction and magnitude. However, other seasonal exposures have also been postulated to drive the seasonality of IMD: surges in IMD activity frequently coincide with or closely follow increases in the incidence of influenza and other respiratory virus infections [10], [11], [12], [13], with elevated IMD risk reported when influenza activity is “epidemic” [14]. This association has led some investigators to suggest that influenza activity may directly influence invasive meningococcal disease risk [10], but evidence for acute effects of changing influenza activity on invasive meningococcal disease risk has to date been limited. This correlated incidence of viral infections and IMD could represent a causal link, but could also represent shared susceptibility to seasonal environmental exposures or seasonal changes in human behavior (e.g., clustering together of individuals indoors due to cold winter weather, holiday gatherings, etc.).

Approaches to the evaluation of links between respiratory viral disease incidence and IMD risk need to be able to adjust for expected crude correlations between multiple seasonally occurring respiratory diseases and environmental exposures. We have previously used regression models with seasonal smoothers, and case-crossover analyses, to evaluate the association between environmental exposures and bacterial respiratory disease [9], [15], [16]. The case-crossover study design provides a means of evaluating causal relationships between rare outcomes, such as invasive meningococcal disease, and brief, transient exposures, such as fluctuating levels of influenza activity, or environmental conditions [9], [16]. We used the case-crossover design, as well as more traditional regression methods, to evaluate the impact of influenza and respiratory syncytial virus (RSV) on meningococcal disease risk in Central Ontario, Canada.

## Methods

**Figure 1:**
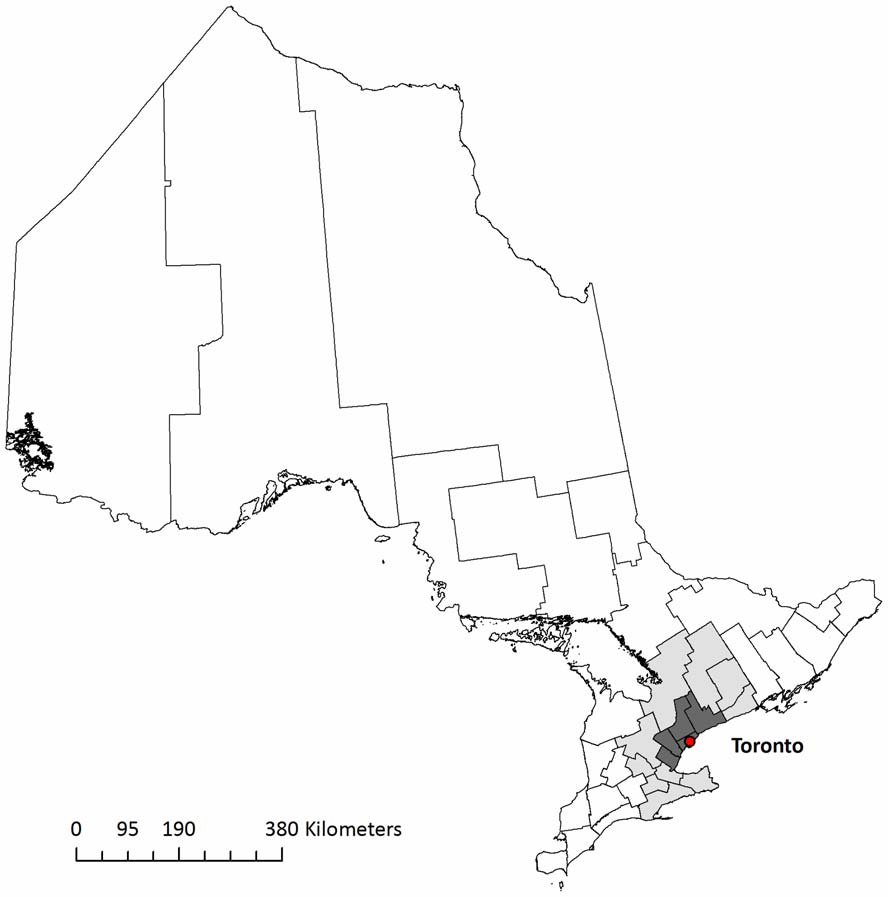
Map of Ontario, Canada, showing Central Ontario and the Greater Toronto Area, as defined in this study.

For the purposes of this study, Central Ontario, Canada, was defined as the Greater Toronto Area (GTA) and contiguous jurisdictions (**Figure 1**). Approximately 68% of the population of the province of Ontario resides within this area, representing over 8.15 million individuals in 2006 [17].

### Case identification

Cases of IMD occurring in Ontario between January 2000 and December 2006 were retrospectively identified through the Ontario Public Health Laboratory System, a provincial reference laboratory that performs all reference testing on meningococcal isolates in the province [3]. Patient characteristics, submission date and disease outcome were ascertained from patient requisitions and electronic databases used for the reporting of notifiable diseases in Ontario: the Reportable Disease Information System (RDIS) (2000–2005) and the integrated Public Health Information System (iPHIS) (2006).

### Exposure estimates

Weekly influenza A and B test volumes and test results (using viral culture or direct antigen detection) for the GTA and RSV virus activity estimates for the province of Ontario were obtained from sentinel laboratories that participate in the Public Health Agency of Canada's Respiratory Virus Detection Surveillance System (RVDSS) for the time period under investigation. The RVDSS is perhaps best known for the support it provides to Canada's “FluWatch” influenza surveillance system [18].

We obtained time series data on ultraviolet radiation and weather from Environment Canada monitoring stations in the GTA (**Table 1**). Where daily readings were taken at multiple locations, the arithmetic means of environmental data were used as exposure variables.

**Table 1:** Sources and measurement sites for exposure variables.

Variable		Measurement site	Source
VIrologic data			Public Health Agency of Canada[18]
	Influenza A and B activity	Greater Toronto Area	
	RSV activity	Ontario	
Weather data		Toronto	Canadian National Climate Archive [36]
	Maximum temperature,°C		
	Mean temperature, °C		
	Maximum relative humidity, %		
	Snowfall, mm		
	Rainfall, mm		
Ultraviolet (UV) data		Toronto	Environment Canada—World Ozone and UV Data Centre [37]
	UV index, per unit change		
	UVA index, per unit change		
	UVB index, per unit change		

### Statistical analyses

We evaluated seasonality of IMD, influenza, and RSV occurrence by calculating autocorrelation coefficients for case counts aggregated at weekly and monthly levels, through spectral decomposition, and by the incorporation of Fourier transform terms into negative binomial regression models [19]. Annual periodicity was not apparent in the IMD dataset, and because data were too skewed for the use of Poisson regression, we modeled underlying trends in IMD occurrence using negative binomial regression models incorporating restricted cubic splines as smoothers. The optimal model was determined based on minimization of Akaike's Information Criterion and incorporated spline knots at six-month intervals [9]. Models incorporated population data derived from the 2001 and 2006 censuses as offsets, with linear interpolation and extrapolation used to generate population denominators for non-census years. The impact of environmental and virological exposures (i.e., level of influenza and/or RSV activity) was assessed by incorporating these terms into models containing spline terms. Exposures were incorporated into models using a backward elimination algorithm, with covariates retained in the final model for *P*≤0.05 [20].

To evaluate acute associations between influenza and RSV activity and occurrence of individual cases, we used a case-crossover approach. This study design is similar to the case-control design, except that it uses self-matching rather than an external control group [21], [22]. This approach reduces confounding by participant characteristics that remain constant over time, while essentially eliminating the risk of selecting unrepresentative controls [21]. A 2∶1 matched-period case-crossover design was used. Hazard periods (person-time during which the event occurred) were defined according to date of symptom onset. Beginning on January 1, 2000, person-time at risk was divided into 3-week time blocks. The two days within each block that could be matched to the hazard period by day-of-week were defined as control days (person-time during which the event of interest did not occur). Each analytic stratum consisted of one case day and two control days. This approach was used to produce random directionality of control period selection, since seasonal or temporal trends may introduce bias when only unidirectional or stereotyped bidirectional control periods are used in the context of exposure data with underlying temporal trends [23].

Given the incubation period for IMD (typically <7 days) [1] we considered exposures occurring within 1 week of case occurrence to fall within plausible effect periods. As respiratory virus infection prevalence was only available as weekly totals, concurrent week respiratory virus exposures were used to estimate effects. Effects were also explored at longer lags (up to 3 weeks prior to case occurrence). We performed both univariable and multivariable analyses (i.e., models restricted to respiratory viruses at single lags, as well as models that included respiratory virus effects at multiple lags and environmental covariates).

Odds ratios for case occurrence, based on influenza or RSV activity, were estimated using conditional logistic regression, with standard errors adjusted for clustering by 3-week time blocks [20]. We explored possible heterogeneity of effect by serogroup or case occurrence during influenza season (November through April) versus non-influenza season using stratum-specific analyses, with heterogeneity of effects across strata assessed using the meta-analytic *Q-*statistic [24]. We explored the possibility of effect modification by age by incorporating multiplicative interaction terms into the regression models.

## Results

### Case Characteristics

Between 2000 and 2006, 240 meningococcal cases were identified in Central Ontario (**Table 2**), with a crude annual rate of 4.4 cases per million population (95% confidence interval (CI): 3.9, 5.0). The median age of cases was 23 years (range: <1 month to 98 years). Risk was not significantly higher in women than in men (incidence rate ratio (IRR)  = 1.27, 95% CI: 0.98, 1.64). The majority of cases were positive for serogroups B (33%) and C (30%). There was an annual trend of decreasing yearly incidence of IMD during the study period (IRR = 0.90, 95% CI: 0.84, 0.97), as reported elsewhere [9].

**Table 2:** Characteristics of cases of invasive meningococcal disease (n = 240) in Central Ontario, Canada, 2000–2006.

Characteristic	No. of Cases (%)
Total	240
Sex	
Male	102 (42.5)
Female	138 (57.5)
Age, years	
0 – 4	46 (19.2)
5 – 9	11 (4.6)
10 – 14	9 (3.7)
15 – 19	29 (12.1)
20 – 24	30 (12.5)
25 – 64	70 (29.2)
≥65	37 (15.4)
Not reported	8 (3.3)
*Neisseria meningitidis* serogroup	
B	80 (33.3)
C	72 (30.0)
W-135	27 (11.2)
Y	58 (24.2)
Other/unknown	3 (1.3)

### Seasonality of Invasive Meningococcal Disease

Case occurrence was significantly increased in the winter months (December, January, and February, mean annualized incidence (cases per million) 5.7, 95% CI: 3.6, 7.8) relative to the autumn (September, October, November, mean annualized incidence (cases per million) 3.1, 95% CI: 2.7, 6.3; IRR = 1.73, 95% CI: 1.20, 2.50). No significant differences in incidence were observed for the spring (mean annualized incidence (cases per million) 4.5, 95% CI: 2.7, 6.3) and summer months (mean annualized incidence (cases per million) 4.4, 95% CI 2.8, 6.0), relative to autumn. Although no clear evidence of periodicity of IMD was observed, either through spectral decomposition or construction of autocorrelograms, evidence of significant annual seasonal oscillation was observed when Fourier transform terms were added to the model (*P*-value for seasonal oscillation with combined sine and cosine terms = 0.013).

**Figure 2:**
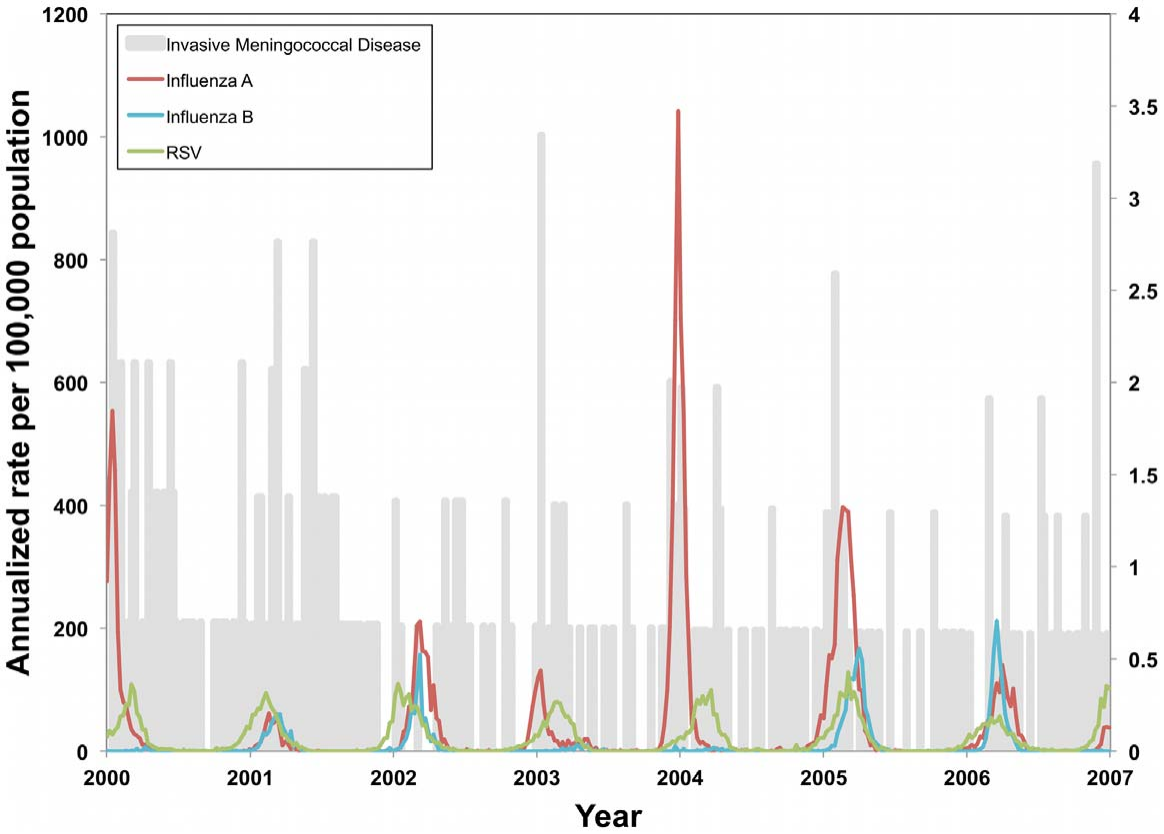
Annualized rates of invasive meningococcal disease (IMD) (right axis) and influenza A, influenza B, and RSV (left axis) between 2000 and 2006. IMD cases are for reported Central Ontario, while influenza A and B activity is reported for the Greater Toronto Area, and RSV activity is reported for the province of Ontario.

Influenza A, influenza B, and RSV all showed strong winter and spring time seasonality, with annual shifts in the timing and height of peak activity during the study period (**Figure 2**). Annual periodicity was observed for RSV, with maximum autocorrelation observed at week 52 (autocorrelation coefficient = 0.71). For influenza B, maximum autocorrelation was observed at week 51 (autocorrelation coefficient = 0.43). Periodicity of influenza A was less regular, with maximum autocorrelation observed at week 60 (autocorrelation coefficient = 0.33). Negative binomial models also showed strong statistical evidence for annual oscillation for all three respiratory viruses (*P* <0.001).

### Negative binomial regression

Univariable negative binomial regression models identified an association between risk of IMD and increasing weekly influenza A activity the week of, and two weeks prior to, case occurrence, and RSV activity during the week of case occurrence only (**Table 3**). Statistically significant associations between several environmental exposures and IMD risk were also identified in univariable models. However, in multivariable models that incorporated cubic spline smoothers, only influenza A activity the week prior to case occurrence was an independent predictor of IMD risk (per 100 case increase, IRR = 1.18, 95% CI: 1.06, 1.31). The effect size associated with RSV activity increased when we included RSV at other lags (IRR 1.81, 95% CI 0.92 to 3.56), though the effect ceased to be statistically significant (P = 0.085), likely reflecting a high-degree of autocorrelation in RSV exposure in the time-series (P≤0.004 for lags of 1, 2, and 3 weeks).

**Table 3:** Variables significantly associated with the occurrence of invasive meningococcal disease in univariable and multivariable negative binomial regression models, Central Ontario, 2000–2006.

Variable	Univariable Models			Multivariable Models with Restricted Cubic Splines		
	IRR	95% CI	*P*-value	IRR	95% CI	*P*-value
Influenza A, per 100 cases						
No lag	1.17	1.06 to 1.29	0.002	---	---	---
1 week lag	1.17	1.07 to 1.29	0.001	1.18	1.06 to 1.31	0.003
2 week lag	1.16	1.04 to 1.28	0.005	---	---	---
RSV, per 100 cases						
No lag	1.27	1.03 to 1.56	0.023	---	---	---
Maximum temperature(°C)	0.98	0.97 to 0.99	0.006	---	---	---
Rainfall (mm)	0.92	0.84 to 0.99	0.031	---	---	---

### Case-crossover analysis

**Figure 3:**
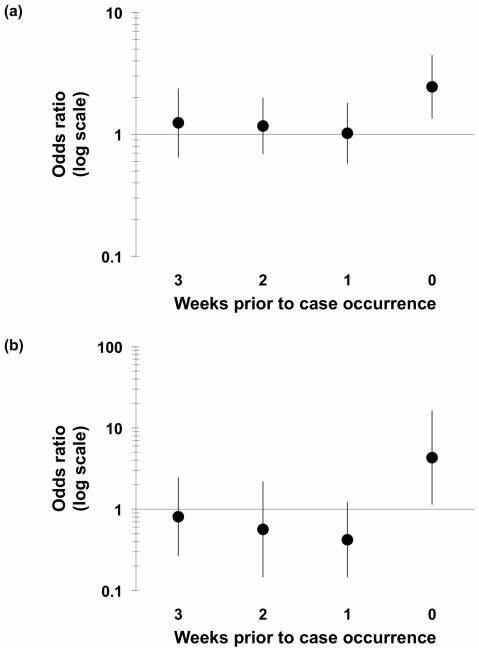
Association between respiratory virus activity and invasive meningococcal disease in Central Ontario, 2000–2006. Odds ratios are plotted on a log scale and are reported per 100 reported cases of (a) influenza A and (b) RSV, while lag times (in weeks) are plotted on the x-axis. An increased risk of IMD was seen with increasing influenza A and RSV activity during the week of case occurrence. Odds ratios are adjusted for respiratory virus activity in the remaining weeks. 95% confidence intervals are indicated by bars.

We estimated effects of respiratory virus activity up to 3 weeks prior to case occurrence (see **Figures S1, S2, and S3** for unadjusted results). Increasing weekly influenza A activity during the week of case occurrence was associated with an acute increase in the risk of invasive meningococcal disease (per 100 case increase, OR = 2.03, 95% CI: 1.28, 3.23; adjusted for previous 3 weeks of influenza A activity, OR = 2.46, 95% CI: 1.34, 4.48) (**Figure 3(a)**). Increasing influenza A activity the week prior to case occurrence was associated with an increased risk of IMD (per 100 case increase, OR = 1.53, 95% CI: 1.03, 2.26), but this effect disappeared after adjusting for influenza activity during the week of case occurrence. In contrast to negative binomial models, increasing RSV activity during the week of IMD case occurrence was associated with increased risk of IMD only after adjusting for RSV activity in prior weeks (unadjusted, per 100 case increase, OR = 1.92, 95%CI: 0.75, 4.93; adjusted for previous 3 weeks of RSV activity, OR = 4.31, 95% CI: 1.14, 16.32) (**Figure 3(b)**). No change in meningococcal disease risk was observed with increasing influenza B activity.

Adjustment for other environmental exposures previously demonstrated to be associated with IMD risk, including ultraviolet radiation [9] and relative humidity [4], [5], [6], [7], [8], [9] did not alter the observed effects. No differences in effect were observed in analyses by serogroup or by influenza season versus non-influenza season. No modification of effect was observed by age group.

## Discussion

The co-seasonality of respiratory viruses and invasive meningococcal disease has long been recognized [13], [25], but establishing the causal nature of this relationship has remained difficult due to the relatively stereotyped late wintertime appearance of both diseases. Using both regression models that incorporate smoothers, and a complementary case-crossover study design, we have demonstrated that increases in influenza A and RSV activity at the population level increase the risk of IMD in Central Ontario even after adjustment for expected temporal trends (in the case of regression models) and matching for seasonal factors (with case-crossover models). By contrast, we identified no association between the incidence of influenza B virus infection and IMD risk.

Our findings reproduce earlier work completed in Denmark and Spain [12], [14]; both of these earlier studies used regression methods with oscillatory seasonal smoothers to demonstrate links between surges in influenza activity and IMD risk after controlling for predictable co-seasonality. Such regression methods require aggregation of case data and exposures, leading to possible “ecological fallacy” [26]. Case-only methods, such as case-crossover design, may reduce distortions caused by data aggregation, and permit analysis of environmental factors associated with case occurrence at the level of the individual [27]. The “self-matching” characteristic of the case-crossover design also reduces confounding by season. For example, in our study, case periods are matched to control periods that are 7–14 days away in time and matched by day-of-week; as such, our estimates would not be confounded by seasonal factors (e.g., school attendance) that would not vary over short time periods.

Our case-crossover findings were consistent with effects identified using regression models (i.e., increased risk of IMD with increased influenza A and RSV activity), and do not appear to be attributable to variation in environmental exposures (such as UV radiation) which we adjusted for in our analyses. We note that our findings with RSV were somewhat less clear-cut than those seen with influenza, which may reflect a higher degree of autocorrelation in the RSV time series than in the influenza time series, with resultant distortion of effects when exposures at multiple lags were introduced into models.

Understanding the mechanisms through which influenza and RSV alter the epidemiology of IMD will have important implications for disease control. Viral infection of the upper respiratory mucosa has been proposed to enhance adherence of *N. meningitidis* to respiratory epithelium [28], [29]. In vitro experimentation has demonstrated enhancement of adhesion of *N. meningitidis* capsule to cultured epithelial cells when influenza A virus neuraminidase is present [29]. RSV-infected cells also appear to have enhanced binding to *N. meningitidis* (and *H. influenzae,* another important cause of bacterial meningitis) to a larynx-derived cell culture [28]. By contrast, Read and colleagues failed to find any effect of experimental infection with influenza B viruses on interaction between *N. meningitidis* and respiratory epithelium [30]. Interestingly influenza B incidence (in contrast to influenza A and RSV incidence) was not associated with changes in IMD risk in our study, although this could have been due to issues of statistical power, as influenza B incidence was lower than incidence of influenza A in our time series.

If the findings reported here can be confirmed, they have important implications for disease control policy. While vaccination against IMD appears to have resulted in important reductions in morbidity and mortality in the province of Ontario [3], the most commonly invasive serogroup in the province is now B, which is not incorporated into the recently introduced quadrivalent vaccine (although promising vaccines against serogroup B are in development [31]). Our findings suggest that influenza prevention via vaccine programs may provide an additional indirect benefit to the province by reducing the risk of IMD in some individuals. Targeting of influenza vaccination at younger individuals in the context of both seasonal and pandemic influenza has been identified as an approach that is possibly more effective at preventing morbidity than direct targeting of older individuals, who are less likely to develop a protective immune response to vaccine [32], [33], [34]. Our observations suggest that an additional indirect benefit of vaccinating younger individuals could include reduction of IMD risk. While the environmental nature of influenza circulation in the community, as represented in this study, makes calculation of attributable risk complex, the ubiquitous nature of influenza suggests that the influenza-attributable fraction of IMD, which would be the fraction of IMD cases that can be eliminated by eliminating influenza transmission, is likely to be high, even for modest elevations in relative risk [35].

Like any observational epidemiological study relying on surveillance data, our study has limitations. Our case-crossover approach serves only as a partial remedy to issues of ecological fallacy related to data aggregation, as our influenza and RSV exposures are available at the weekly, rather than daily, level. Furthermore, respiratory viral activity levels are derived from a provincial system, whereas IMD cases reside only in the central part of the province. This latter issue is not likely to serve as an important source of bias, as most of Ontario's population resides in the central part of the province. Finally, due to the aggregated nature of influenza exposure data, we are unable to define a precise effect period that might provide insights into whether this effect is driven by increased transmission, increased propensity for invasive disease, or increased colonization, though biological experiments described above strongly suggest that the latter may be important.

In summary, we identified strong associations between influenza and RSV activity and risk of invasive meningococcal disease in the Canadian province of Ontario. These effects persisted after adjusting for coincident environmental factors and using both smoothers and a case-crossover approach to minimize confounding. Such effects are biologically plausible, and have important implications for development of vaccines and disease control policy targeted at reducing the burden of these important infectious diseases.

## Supporting Information

Figure S1 Association between influenza A activity and invasive meningococcal disease. Crude odds ratios per 100 cases of influenza A are shown for the week of IMD case occurrence (week 0) and for lags of up to 3 weeks prior to case occurrence. Odds ratios are not adjusted for influenza A activity in the remaining weeks and are plotted on a log scale. Lag times (in weeks) are plotted on the x-axis. 95% confidence intervals are indicated by bars.
(TIF)

Figure S2 Association between influenza B activity and invasive meningococcal disease. Crude odds ratios per 100 cases of influenza B are shown for the week of IMD case occurrence (week 0) and for lags of up to 3 weeks prior to case occurrence. Odds ratios are not adjusted for Influenza B activity in the remaining weeks and are plotted on a log scale. Lag times (in weeks) are plotted on the x-axis. 95% confidence intervals are indicated by bars.
(TIF)

Figure S3 Association between RSV activity and invasive meningococcal disease. Crude odds ratios per 100 cases of RSV are shown for the week of IMD case occurrence (week 0) and for lags of up to 3 weeks prior to case occurrence. Odds ratios are not adjusted for RSV activity in the remaining weeks and are plotted on a log scale. Lag times (in weeks) are plotted on the x-axis. 95% confidence intervals are indicated by bars.
(TIF)
