# Supplementary figures and images for: Respiratory Virus Infection and Risk of Invasive Meningococcal Disease in Central Ontario, Canada
Source: PLoS One. 2010 Nov 17;5(11):e15493. doi: 10.1371/journal.pone.0015493 (PMC2984510; doi:10.1371/journal.pone.0015493)

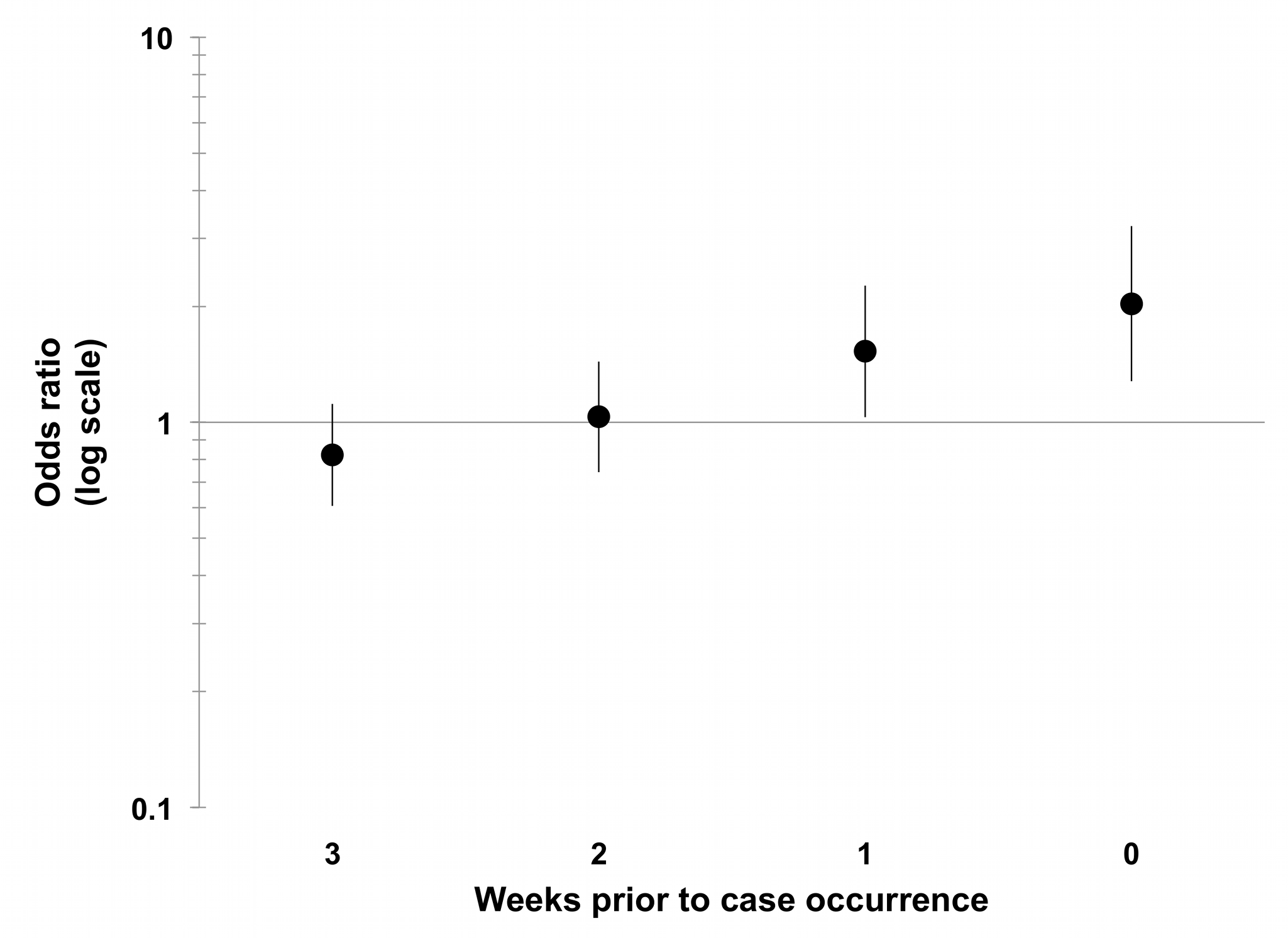

Supplement: Figure S1 — Association between influenza A activity and invasive meningococcal disease. Crude odds ratios per 100 cases of influenza A are shown for the week of IMD case occurrence (week 0) and for lags of up to 3 weeks prior to case occurrence. Odds ratios are not adjusted for influenza A activity in the remaining weeks and are plotted on a log scale. Lag times (in weeks) are plotted on the x-axis. 95% confidence intervals are indicated by bars. (TIF) [file pone.0015493.s001.tif]

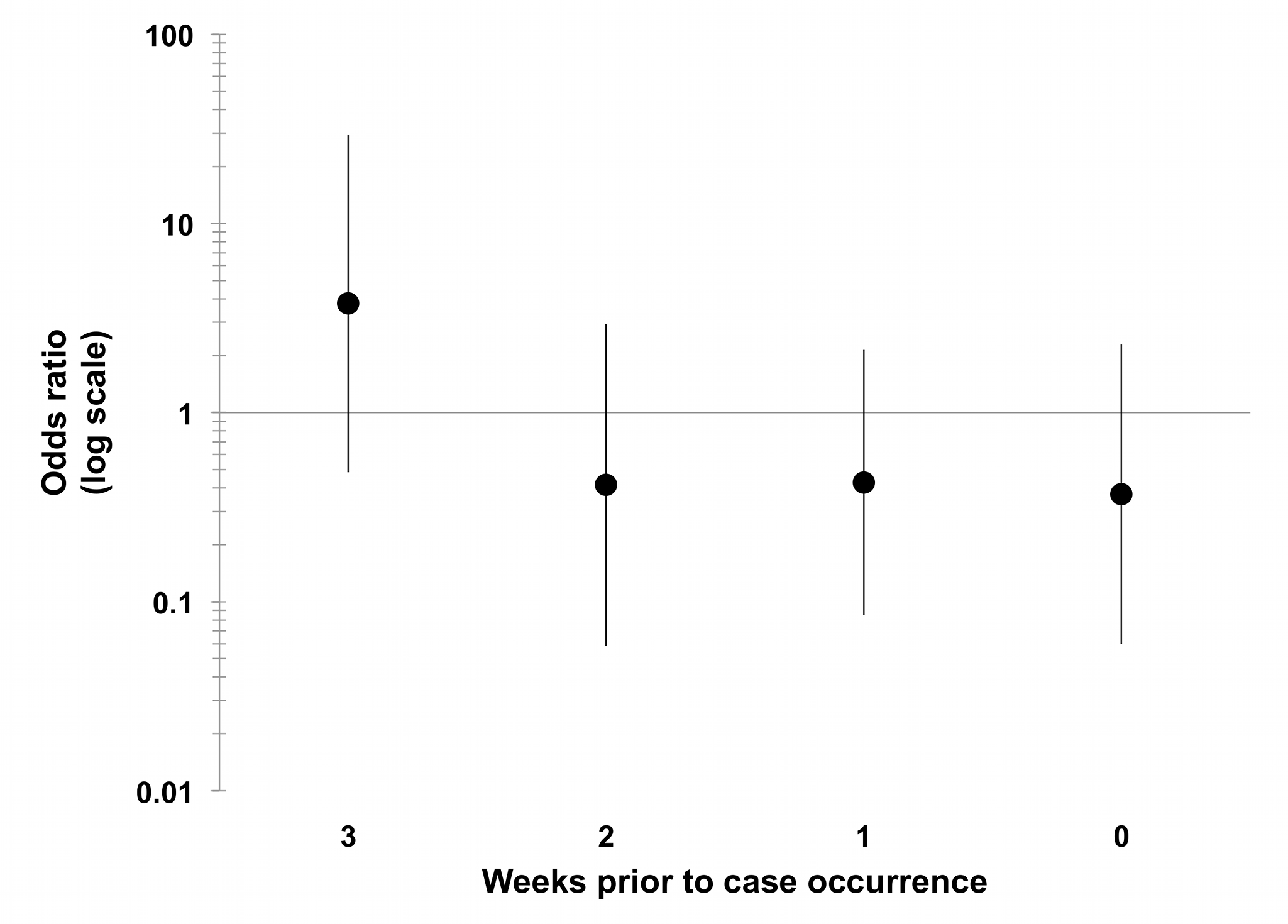

Supplement: Figure S2 — Association between influenza B activity and invasive meningococcal disease. Crude odds ratios per 100 cases of influenza B are shown for the week of IMD case occurrence (week 0) and for lags of up to 3 weeks prior to case occurrence. Odds ratios are not adjusted for Influenza B activity in the remaining weeks and are plotted on a log scale. Lag times (in weeks) are plotted on the x-axis. 95% confidence intervals are indicated by bars. (TIF) [file pone.0015493.s002.tif]

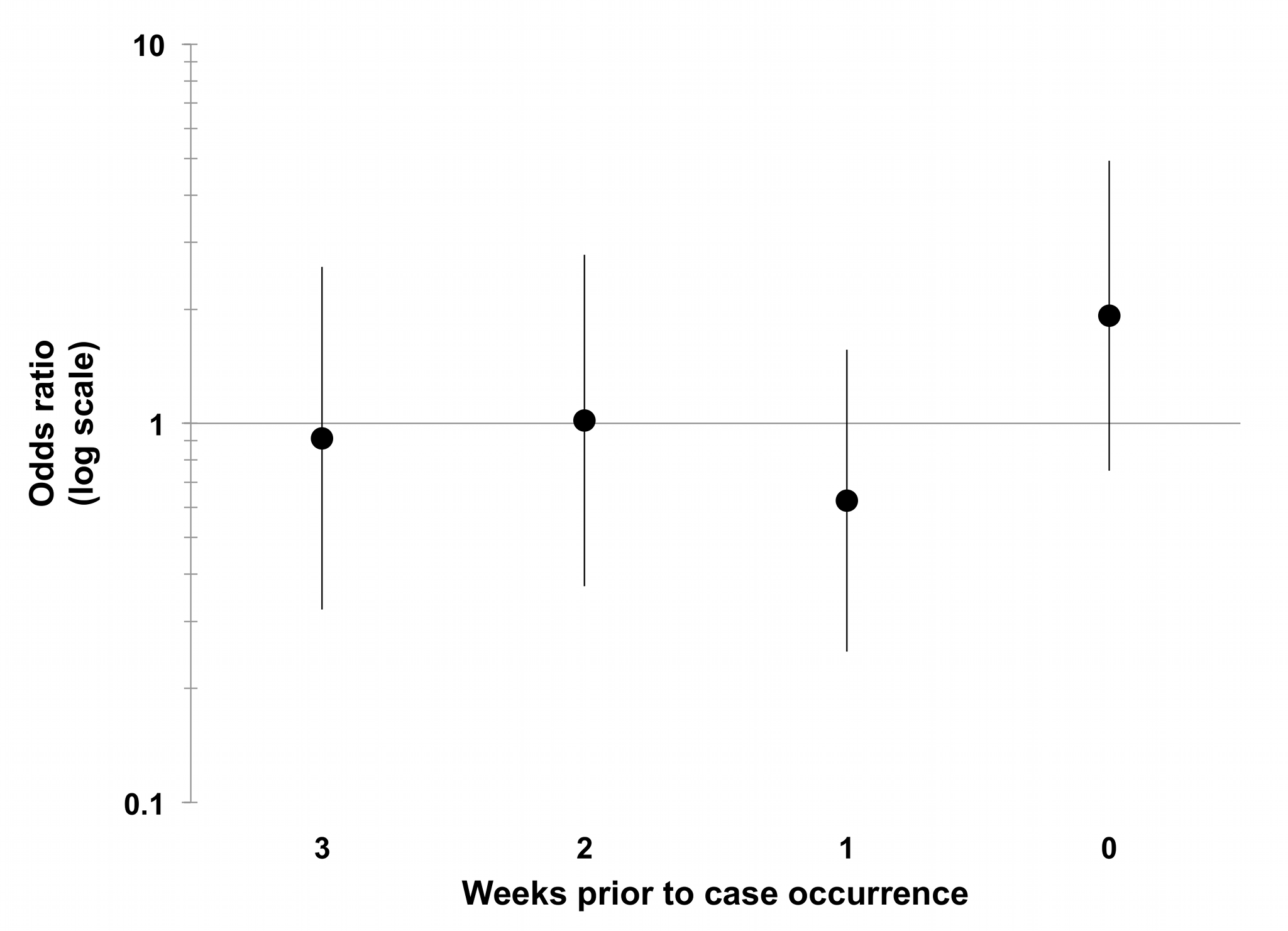

Supplement: Figure S3 — Association between RSV activity and invasive meningococcal disease. Crude odds ratios per 100 cases of RSV are shown for the week of IMD case occurrence (week 0) and for lags of up to 3 weeks prior to case occurrence. Odds ratios are not adjusted for RSV activity in the remaining weeks and are plotted on a log scale. Lag times (in weeks) are plotted on the x-axis. 95% confidence intervals are indicated by bars. (TIF) [file pone.0015493.s003.tif]
